# Use of digital periodontal data to compare periodontal treatment outcomes in a practice-based research network (PBRN): a proof of concept

**DOI:** 10.1186/s12903-020-01284-3

**Published:** 2020-10-28

**Authors:** Stefanie Anna Peikert, Felix Mittelhamm, Eberhard Frisch, Kirstin Vach, Petra Ratka-Krüger, Johan Peter Woelber

**Affiliations:** 1 Department of Operative Dentistry and Periodontology, Faculty of Medicine, University of Freiburg, Hugstetter Straße 55, 79106 Freiburg, Germany; 2 Hamburg, Germany; 3 Hofgeismar, Germany; 4 Department of Medical Biometry and Statistics, University Freiburg Medical Center, Stefan-Meier-Straße 26, 79104 Freiburg im Breisgau, Germany

**Keywords:** Practice-based research network (PBRN), Periodontology, Periodontal therapy, Outcomes

## Abstract

**Background:**

Scientific studies in dentistry are mainly conducted at universities. However, most patients are treated in dental practices, which differ in many ways from treatment at the university. Through the establishment of practice-based research networks, however, it is also possible to examine studies in a real-world setting in dental practices. For this reason the aim of this non-interventional, observational study was to develop and evaluate a digital procedure to access, extract and analyse recorded clinical data in practices to assess periodontal treatment outcomes.

**Methods:**

Participating periodontists were former or active postgraduate students of a master’s course in periodontics in Freiburg who routinely used a digital periodontal diagnostic program. All available stored periodontal patient charts were extracted, anonymized and digitally sent to the study centre.

**Results:**

In this study, data were collected from 6301 patients from 9 different practices. Information such as probing depth (PD), bleeding on probing (BOP), mobility, furcation and gingival attachment for 153,163 teeth at first visit were successfully transferred to the study centre. During the average observational period of 9.77 years, only 2.8% of all teeth were lost. The number of visits was significantly negatively correlated with BOP (*p* < 0.0001), and the number of BOP-positive sites was significantly correlated with deeper PDs (*p* < 0.001).

**Conclusion:**

The presented procedure was able to gather a large amount of practice-based periodontal data, and thus this study may support practice-based research networks. The data indicate that systematic and supportive periodontal therapy is successful on a practice-based level.

*Trial registration* The study was internationally registered on 4 January 2017 in the German Clinical Trials Register (DRKS 00011448). https://www.drks.de/drks_web/navigate.do?navigationId=trial.HTML&TRIAL_ID=DRKS00011448

## Background

Clinical periodontal research mainly takes place in academic facilities, where treatment is usually carried out by highly trained staff members [1]. However, according to data from the US, these facilities treat less than 1% of the total population [2]. Accordingly, 99% of the patients receive their dental care in private practices and in an environment that differs greatly from that in universities with regard to treatment time, costs, education and payment of caregivers, and use of evidence-based treatment protocols [3]. To understand the difference in treatment between an academic setting and private practice, the idea of practice-based research networks (PBRNs) was introduced in the US in the early 1970s [4]. The aim of PBRNs was to quickly bring both scientific advantages into practice and practice-relevant topics onto the research agenda [4]. Therefore, improved communication between practice and research may lead to better patient care [5–7].

The digitization of dental practices offers new possibilities for research on a practice-based level. Historically, the healthcare system has generated large amounts of records for patient care, compliance and regulatory requirements, with most of these data stored as hard copies [8]. For example, in Germany, most private practices use software to record relevant periodontal data for their patients for documentation. Hence, these records can also be used for scientific investigations and digitally collected to support a practice-based research network. Worldwide, a few studies have already been carried out in which electronic dental record data from practice-based research network practices have been examined with regard to assess dental treatment outcomes [9–12]. For example, electronic data from a dutch practice based research network, which consisted of 24 dental practices, was collected and examined with regard to the survival rate of direct restorations. It could be shown that there was a satisfactory longevity of the restaurations, although there were substantial differences in outcome between practitioners [11]. In periodontology, too, new program software makes it possible to digitally collect data on periodontal patients from different dental practices to analyse it with regard to a scientific question. One of the most commonly used software tools in Germany is the program Parostatus® (Parostatus.de GmbH, Berlin, Germany). The software is accredited by the standards of the German Periodontal Society [13]. The program includes the monitoring of several periodontal treatment parameters, such as pocket depth (PD), bleeding on probing (BOP), mobility, furcation involvement and gingival recessions.

To the best of the authors’ knowledge, no studies to date have attempted to digitally collect data on periodontal patients from different dental practices. Therefore, the aim of this study was to develop a procedure to digitally extract periodontal data from specialized periodontal practices to support the concept of digital extraction of data, analyse the collected data of treatment outcomes, and compare the findings with current literature.

## Materials and methods

### Recruitment

Active and former postgraduate students of a master’s course in periodontics and implant therapy at the University of Freiburg, Germany [14] were contacted via e-mail in January 2017. All of them have been taught a standard protocol according to approved guidelines to ensure a similar level of treatment and education [15]. The training program thus includes the components of systematic treatment of periodontal disease (initial therapy, surgical periodontal therapy and risk-associated maintenance phase (supportive periodontal therapy/ SPT)) [15, 16]. Each participant was informed about the structure and the study design of this non-interventional, observational trial and signed a consent form. To participate in the study, it was required that the software Parostatus® be used in the practice on a daily basis.

### Digital procedure

It was confirmed that all the participants used the software Parostatus® on a daily basis to record data from periodontal examinations. The software company Parostatus® itself provided special additional software to extract these recordings anonymously. First, the program generated a list of all the recorded patients in which full periodontal status was established for analysis. Within the full-mouth periodontal status, probing depths were gathered on 6 sites per tooth, as was bleeding on probing (BOP) 30 s after probing [17]. Moreover, gingival recessions and clinical attachment level were determined. Furthermore, mobility level and furcation defects were measured [15, 18, 19]. Furcations were measured on multi-rooted teeth with the aid of the Nabers probe according to the Hamp, Nyman and Lindhe classification [18]. All patients undergoing a periodontal treatment period over at least one year, regardless of initial periodontal status, were screened for inclusion eligibility. Patients revealing a full periodontal data set were included, patients with incomplete data sheets or redundant data (no complete full-mouth periodontal status as defined above) were excluded. Moreover to analyse a change in the values over the course of the recorded visits, only the first 8 visits were used, as the majority of practices provided no further data beyond this number of visits. The type and extent of periodontal treatment of individual patients was not part of the data evaluation.

Furthermore, only patients with > 1 visit were counted to analyse the change in BOP. Tooth loss was calculated between the first visit and the last visit.

Following submission, the program automatically anonymized all the chosen patients. The program generated five files in.csv format (Comma-separated values or more rarely Character-separated values) by clicking on the corresponding field: the standard pocket depth (ST.csv), furcation (Furka.csv), tooth mobility (Bewegl.csv), gingival recession (Gingivaverlauf.csv) and the Bleeding on Probing (BOP.csv) are each generated as a single file. The generated files needed to be saved and copied into an e-mail.

The e-mail containing the anonymous patient data was sent to a principal investigator at the University of Freiburg. The participating practices were pseudonymized, and the anonymous data were sent to a second investigator. The data sheets were checked for completeness and sent to the Institute of Medical Biometry and Statistics of the University of Freiburg for statistical evaluation.

### Observations of the study centre

After data transfer, the dentists answered five questions evaluating the perceived quality of the digital procedure, the time required and their willingness to continue participation in the study.

### Statistical procedures

For descriptive analyses, the mean, median, and standard deviation were computed for every outcome. One-way ANOVA and Kruskal–Wallis tests were used to check for differences between the practices regarding different outcome variables. Pearson correlation coefficients were computed to show correlations among several variables. A linear mixed model was used to estimate the influence of tooth position on the PD value. The calculations were performed with the statistical software STATA 14.2. (StataCorp LLC, Texas, USA). The level of significance was set at *p* < 0.05.

## Results

### Demographic data

In total, data from 6301 patients from 9 practices were extracted and analysed. The mean patient age per practice was 55.84 years. The average recording time of the different practices was 9.77 years of records. Moreover, how frequently the patients attended the practices was also analysed. The average time between two visits per practice in years was 1.94 ± 2.16. The results are shown in Table 1. During the recorded treatment period, patients attended a practice 4.4 times on average. Table 2 summarises the number of visits for all patients per practice.

**Table 1:** Patients recruited in the participating nine practices

Practice	n	Average age in years	Recorded years	Average time between visits in years
1	70	47.45 [1.00–78.01]	2.09	0.17 ± 0.29
2	409	56.98 [19.97–86.00]	5.15	1.05 ± 1.21
3	1822	56.01 [7.03–90.02]	5.63	0.85 ± 1.28
4	67	56.95 [32.01–87.05]	2.78	0.25 ± 0.39
5	356	56.04 [14.99–82.96]	9.25	1.77 ± 2.06
6	536	56.98 [15.95–88.93]	13.43	1.47 ± 2.37
7	776	54.02 [14.02–91.00]	9.77	0.92 ± 1.34
8	2011	54.07 [3.96–91.94]	18.34	2.65 ± 2.30
9	254	59.50 [21.00–87.90]	3.54	0.58 ± 0.78
Total	6301	55.94 [1.00–91.94]	9.77	1.94 ± 2.16

n = number of treated patients, average age of the patients in years (median [range]), recorded years, average time between visits in years (mean ± SD (standard deviation))

**Table 2:** n = number of visits for all patients per practice with median [range] and mean ± SD (standard deviation)

Practice	n visits	Median [range]	Mean ± SD
1	112	1 [1–7]	1.70 ± 1.15
2	1161	2 [1–33]	3.10 ± 3.49
3	3324	2 [1–45]	2.68 ± 4.08
4	107	1 [1–4]	1.52 ± 0.75
5	1151	2 [1–9]	2.79 ± 1.77
6	1043	1 [1–11]	2.09 ± 1.60
7	2406	2 [1–18]	3.10 ± 2.44
8	12,485	5 [1–29]	5.79 ± 4.52
9	457	1 [1–5]	1.62 ± 0.78

### Presence of teeth in the investigated population

Overall, 153 163 teeth were recorded at the first visit. 83.97% of this cohort had a least 20 functional teeth. The teeth 18 (0.9%), 28 (0.8%), 38 (1.2%) and 48 (1.2%) made up the smallest percentage of all recorded teeth and were accordingly the most missing teeth.

### Bleeding on probing (BOP)

Together, all of the teeth had a positive BOP score of 21.8%. Tooth 28 presented the highest mean value, with 30.45%, while the lowest mean value was calculated for tooth 31, with 15.15%. Molar teeth showed the highest values, followed by premolars, canines and incisor teeth. The calculated average value of all 6 positions resulted in a mean value for every single tooth, which is shown in Table 3. The mean starting value of BOP was significantly different in every practice (*p* < 0.001). The more often a patient attended the practice, the lower the BOP was (r = − 0.29; *p* < 0.0001).

**Table 3:** Collected data of the patients’ 1st visits

Tooth	n	Tooth frequency in %	BOP in %	Mean PD	SD PD
11	5569	3.6	18.27	2.44	1.67
12	5468	3.6	18.98	2.45	1.65
13	5881	3.8	19.05	2.53	1.71
14	4924	3.2	22.09	2.70	1.77
15	4902	3.2	23.21	2.77	1.75
16	4487	2.9	28.37	3.10	1.95
17	4568	3.0	29.81	3.28	2.10
18	1382	0.9	30.18	3.15	2.15
21	5555	3.6	17.78	2.45	1.65
22	5465	3.6	18.65	2.41	1.61
23	5887	3.8	19.05	2.51	1.66
24	4961	3.2	22.77	2.72	1.75
25	4849	3.2	24.15	2.77	1.75
26	4396	2.9	29.26	3.11	1.97
27	4467	2.9	31.18	3.30	2.10
28	1301	0.8	30.45	3.12	2.15
31	5825	3.8	15.15	2.11	1.45
32	5983	3.9	15.83	2.19	1.49
33	6184	4.0	16.8	2.32	1.54
34	5694	3.7	18.58	2.49	1.60
35	5292	3.5	21.31	2.66	1.69
36	4026	2.6	28.7	3.11	1.91
37	4611	3.0	30.05	3.32	2.02
38	1772	1.2	27.21	3.32	2.12
41	5810	3.8	16.00	2.13	1.46
42	5952	3.9	16.84	2.21	1.51
43	6174	4.0	17.71	2.31	1.54
44	5733	3.7	19.40	2.47	1.57
45	5362	3.5	20.98	2.64	1.66
46	4124	2.7	27.73	3.07	1.89
47	4738	3.1	29.92	3.28	2.03
48	1821	1.2	29.06	3.34	2.14
Total	153,163		22.95	2.66	1.78

n = number of teeth, tooth frequency in %, BOP in % per tooth, mean PD = mean pocket probing depth, and SD PD = standard deviation of PD

The table for the BOP of each tooth can be found in the Additional file 1 (see Additional file 1: Table S3).

As an additional question, the relation between BOP and PD could be analysed for 96,194 teeth. It could be found that as more sites of a tooth had a positive BOP, the deeper its probing depths (*p* < 0.001) (Table 4).

**Table 4:** Relation between BOP and PD

BOP (sites per tooth)	n	Mean PD (mm)
0	56,828	2.06 (1.54)
1	9957	3.03 (0.95)
2	8256	3.28 (1.03)
3	6621	3.34 (1.07)
4	5562	3.74 (1.13)
5	3122	4.00 (1.19)
6	5848	4.45 (1.42)

BOP = bleeding on probing; n = number of teeth, mean PD = mean values of pocket depth with standard deviation (in parentheses)

### Pocket depth (PD)

**Fig. 1:**
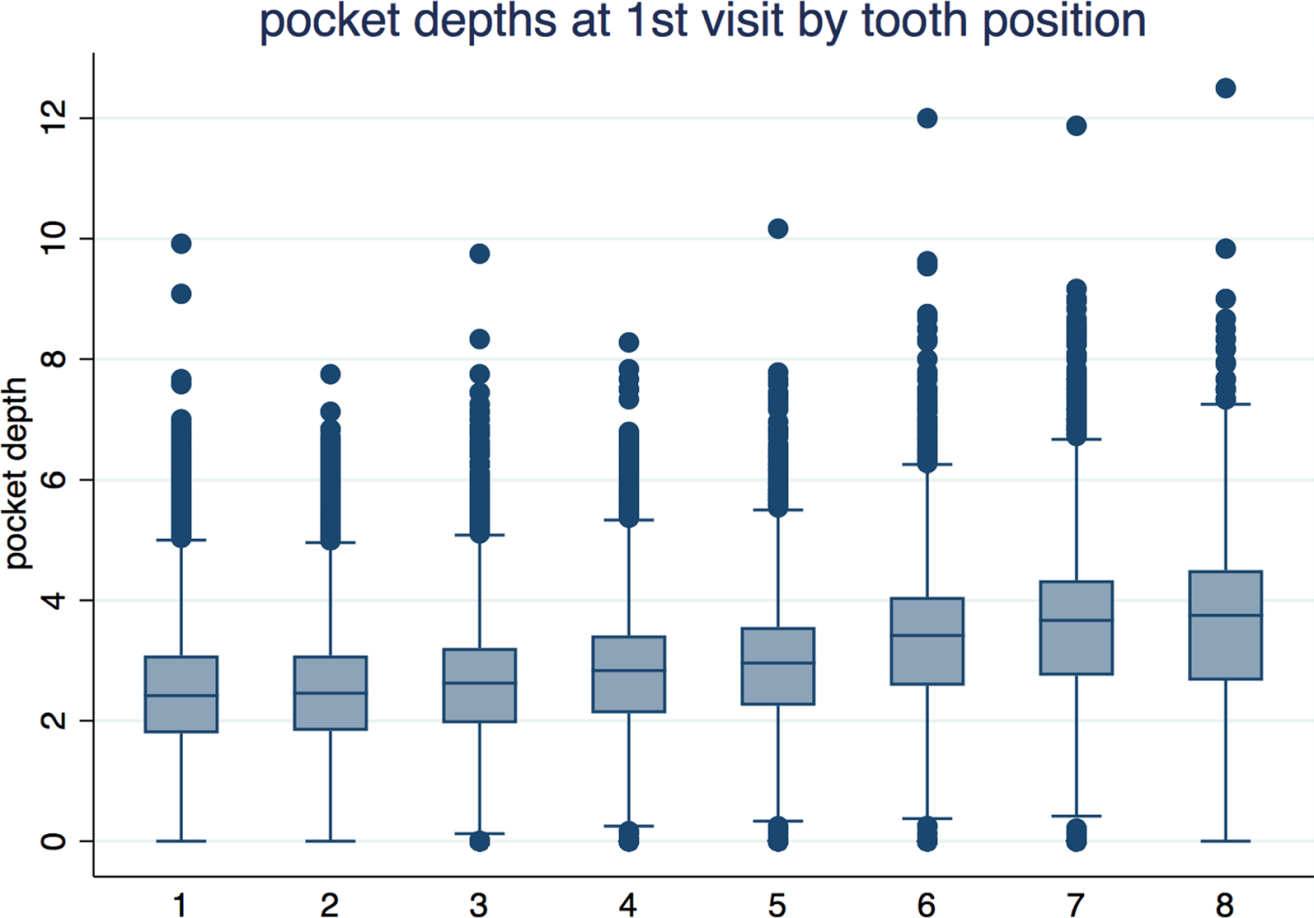
Pocket depths at 1st visit by tooth position

Table 3 presents the mean pocket depth for each tooth position at the first visit. The spreadsheet was translated into a graph (Fig. 1). The tooth position from anterior to posterior showed a significant positive correlation on the PD value (*p* < 0.001). Moving from anterior to posterior in tooth position, an increase in the PD value of 0.18 mm per tooth was detected.

**Fig. 2:**
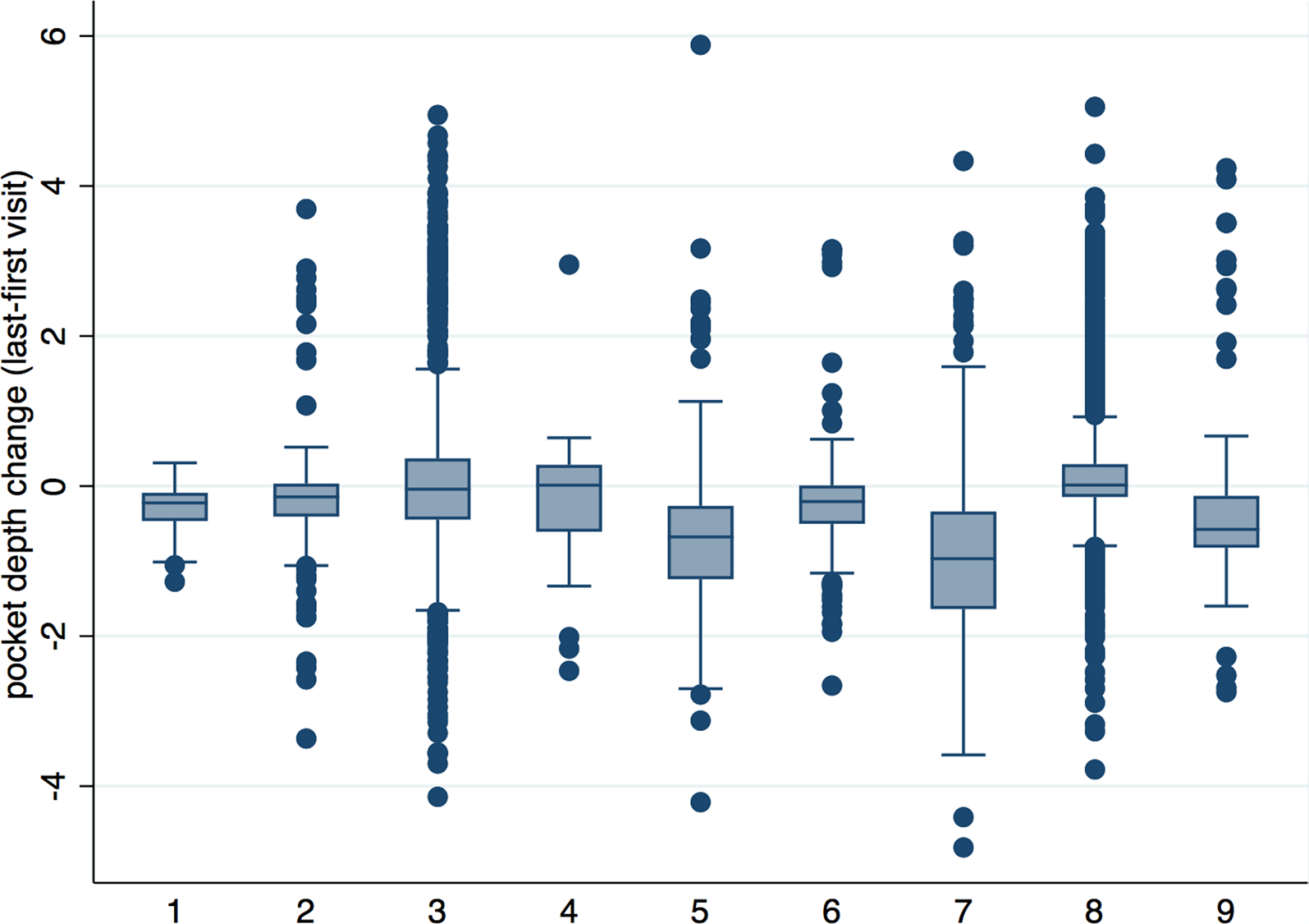
Change of pocket depth between first and last visit

Regarding the change of the pocket depths, 7 of the 9 practices were able to reduce the mean PD with the exception of practices 3 and 8. When comparing the first and last visit, practice 3 and practice 8 showed a slight increase in pocket depths (Fig. 2).

### Furcation

A wide range was found (*p* < 0.001) in the furcation involvement between the practices at the first visit. Maxillary molars with furcation involvement were found in 18.89% at the first visit and 22.02% at the last visit. Mandible molars were only found in 12.83% at the first visit and 15.15% at the last visit.

### Mobility

Mobility decreased significantly over the observed period (*p* < 0.0001). However, the decrease was not within a clinically relevant range (first visit mean mobility 1.29 vs. last visit mean mobility 1.28). The average tooth mobility over the treatment period is summarized in the Additional file 1 (see Additional file 1: Tables S1 and S2).

### Tooth loss

Table 5 summarizes the probability of tooth loss depending on the position of the tooth. The most frequently lost tooth over the treatment period was tooth 28 (19.72%). On average, only 2.8% (standard deviation = 16.58%) of the teeth were extracted or lost for other reasons.

**Table 5:** Probability of tooth loss (in percent) depending on tooth position

Tooth	18	17	16	15	14	13	12	11	21	22	23	24	25	26	27	28
%	14.98	5.57	3.86	3.42	2.72	1.30	1.56	1.71	1.58	1.63	1.18	2.92	3.68	4.17	6.00	19.72
%	12.20	4.17	4.31	1.95	1.29	0.54	0.88	1.06	1.11	1.19	0.41	1.77	2.34	4.04	3.67	12.66
Tooth	48	47	46	45	44	43	42	41	31	32	33	34	35	36	37	38

### Observations of the study centre

#### Practicability of the procedure

Overall, the response to the procedure was positive. Practices 1, 2 and 5 felt that the procedure was simple, fast and straightforward. Practice 3 reported initial problems with the installation of the program while practice 6 had difficulties generating the.csv files. Practices 7 and 8 gave no response to the questions.

#### Participation in further studies

Five practices agreed to participate in further studies, and 3 practices did not respond. No further information was given regarding any changes to the procedure.

#### Compensation/motivation for the participating practice

No answers were given from practices 4, 7 and 8. One practice cited their friendly relationship with one of the investigators as their motivation for participation. Two practices were interested in “benchmarking” to compare their treatment outcomes with those of other practices. Two practices were interested in obtaining a certificate stating that the practice was part of a PBRN. One practice was simply interested in the research results, while another practice wanted to be named in a scientific publication.

## Discussion

The aim of this study was to evaluate a digital procedure to extract periodontal-related patient records from private practices and to analyse the extracted data to support a practice-based research network.

The average patient age across all 9 practices was 55.84 years, which was older than the average in other previous long-term retrospective studies, such as Carnevale et al. [20, 21] with 53 years, Eickholz et al. [22] with 46.6 years, and König et al. [23] with 46 years.

In regard to the supportive periodontal treatment (SPT), the average number of visits per patient was found to be 2.71 over the recorded time. The average time between the two visits per practice in years was 1.94 ± 2.16. Therefore, for all the practices, an average patient visited only once in two years. The reasons for this comparable low grade of recall may be multifactorial. One factor could be financial, because health insurance companies in Germany only pay for an extensive subgingival instrumentation once every two years. It is up to the individual patient to pay out of pocket for any further periodontal examination advised by the dentist. Nevertheless, with the introduction of periodontal risk assessment by Lang and Ramseier, intervals can be performed according to this scheme [24]. According to this scheme, the recall interval can be recommended between one and four times a year on an individual basis [25]. Another reason to consider for longer intervals between patient visits is that patients that see periodontal specialists may also have a referring dentist who carries out the SPT.

The data showed an increased BOP when the patient attended the first visit and a decreased BOP after periodontal therapy, which is in accordance with previous literature [17, 26].

Furthermore, the data revealed a significant improvement of PDs between the first and last visit, which showed that periodontal treatment was effective in the practices. This effect of periodontal therapy is also indicated in prior scientific literature [27–29]. Thus, this evidence from university settings was confirmed by the present practice-based data.

It was also demonstrated that there was a correlation between BOP and PD. Lower PD values could be found with lower inflammation, which is represented by a lower BOP. This correlation was also found in previous university studies [30]. Regarding the process of PD change, all practices showed significant reductions of PD from the first visit to the last visit, except for practices 3 and 8. There may be different reasons for an increase in PD during periodontal therapy in these practices. However, these factors can only be speculated due to the design of this study. Practice 8 is the practice with the longest recorded data set, and a mild deepening of the pocket depth over the years of SPT was also shown in another study [23]. In this study, after 8 years of SPT, the mean PD increased from 2.9 to 3.6 mm after active treatment. In addition, statistical calculations also included values in the healthy range. Thus, PDs of 1 mm might evolve to 3 mm PD without any practical relevance.

It was found that the gingival recessions increased after the therapy relative to the first visit. Subgingival instrumentation often leads to tissue tightening and shrinkage, which is associated with recession formation [31]. Therefore, the measured gingival levels might be higher before therapy compared to after therapy, which is supported by a prior university study [32].

Molars with furcation involvement in the maxillary could be found in 18.89% of cases at the first visit and 22.02% at the last visit, whereas molars in the mandible could only be found in 12.83% at the first visit and 15.15% at the last visit. This is in line with research by Svärdström and Wennström [33], which described that molars in the maxilla more frequently showed a furcation than molars of the mandibular. Regarding tooth mobility, a negative correlation was found between mobility and number of visits. Giargia and Lindhe [34] described in a review that a reduction of periodontal inflammation also resulted in reduced mobility, which was confirmed by the results of this study.

Wisdom teeth in all quadrants showed the highest percentage of tooth loss, followed by the remaining molars and the premolars. Tooth loss in the maxilla was found more often than in the mandibular. Similar findings are published in the scientific literature [35–37]. Altogether only 2.8% (standard deviation = 16.58%) of the teeth were extracted or lost for other reasons in the average recording time of 9.77 years. In another study conducted in Germany with data of a major German national health insurance company, an average tooth loss of 46.2% was determined after periodontal treatment in the investigation period of 4 years [38]. This indicates that a specialization in periodontology, such as received by the dentists in our network of practices, could contribute to a higher rate of tooth survival.

### Limitations

The main limitations of this study lie in the lack of precise information regarding the performed periodontal therapy. In this context, no further statement could be made regarding the periodontal risk factors and the periodontal diagnosis of the patients. The complexity of periodontal therapy, if subgingival instrumentation was performed with or without any additional surgical procedures, cannot be seen from our data. Furthermore, any adjuvant antibiotic therapy also remains unknown [39]. Beyond that, in some practices, the initial therapy and the supportive periodontal therapy might not be coordinated by the dentist but by dental nurses or dental hygienists. Depending on the level of specialization and experience, a wide variety of treatment outcomes can be expected. Pressure applied when probing the tooth pocket or the probing instrument itself are only a few of the factors that can have an influence of the measurement outcome [40, 41]. Moreover, due to the program's features, only periodontal parameters are collected by default. Plaque indices are possible, but are used by the practices in very different ways (e.g. Quigley Hein Index or Approximal Plaque Index according to Lange). Therefore, the extracted dataset contained no information on plaque.

The collected data reflect the dental treatment situation in Germany. Accordingly, the results may only be comparable to other non-industrialized countries.

The examinations carried out within the framework of the study were not carried out by a single dentist, but by a total of 9 different dentists. On one hand, the participating dentists were not calibrated and did not follow a standardized treatment protocol. However, as strength of the study, all participating practices had a special education in periodontology, as they were all former or active postgraduate students of a master’s degree course in periodontology [14]. The results might be different when data are collected from general dental practices with no specific interest in periodontology or by a single examiner.

Furthermore, the main population is in a special range of age (55.84 years). Should further practices participate in future studies, this will result in an even more representative number with regard to the average age of patients requiring periodontal treatment.

All in all, the practice-based research network and the associated digital programme have great potential for further investigations in which other factors such as treatment costs or treatment types can be analysed. Future projects should try to improve the amount and quality of data recording. The software program should record, in addition to the periodontal data, general health issues and more specific data regarding the therapy (e.g., antibiotics, regenerative treatments, etc.). One fundamental principle of this method should be to not interfere with the daily routine of the practitioner, as dental practices are dependent on economical and efficient processes [42]. The results of this study were mostly positive regarding the applied procedure. Participating practices 1, 2, 3 and 5 found the procedure to be unproblematic, logical, simple and fast.

Furthermore, the lack of standardization in comparison to studies from a research facility must be compensated for by a high number of participating practices. Therefore, the practice-based research network should be extended to improve the data quality and to obtain a representative idea of the periodontal care situation.

## Conclusion

The described process to extract periodontal data from practices was able to deliver a high number of periodontal patient records even with a relatively low number of periodontal practices. The collected data set supported several positive university studies of periodontal therapy (such as reduction of bleeding on probing and probing depths and a low number of tooth loss during supportive periodontal therapy) and side-effects of periodontal disease or therapy (increase of gingival recession) on a practice-based level. Furthermore, the data set showed a certain variety of data in the different practices, which provides the opportunity to use the process as a feedback tool for the participating private practices.

## Supplementary information

**Additional file 1: Table S1**. Collected data of the first visit of the patients regarding tooth mobility. **Table S2**. Collected data of the last visit of the patients regarding tooth mobility. **Table S3**. Average Bleeding on probing (BOP) of each tooth.

## Data Availability

The data that support the findings of this study are available on reasonable request from the corresponding author.

## Abbreviations

BOP: Bleeding on probing
PBRN: Practice-based research network
PD: Pocket depths
SPT: Supportive periodontal treatment
