# Supplementary material for: Use of digital periodontal data to compare periodontal treatment outcomes in a practice-based research network (PBRN): a proof of concept
Source: BMC Oral Health. 2020 Oct 28;20:297. doi: 10.1186/s12903-020-01284-3 (PMC7594469; doi:10.1186/s12903-020-01284-3)
Supplement: Supplementary file 1 — Additional file 1: Table S1. Collected data of the first visit of the patients regarding tooth mobility. Table S2. Collected data of the last visit of the patients regarding tooth mobility. Table S3. Average Bleeding on probing (BOP) of each tooth. [file 12903_2020_1284_MOESM1_ESM.docx]

Additional file 1

Table S1: Collected data of the first visit of the patients regarding tooth mobility

| **Tooth** | **n** | **median** | **mean** | **sd** |
| --- | --- | --- | --- | --- |
| 11 | 887 | 1 | 1.28 | .50 |
| 12 | 680 | 1 | 1.29 | .50 |
| 13 | 365 | 1 | 1.31 | .55 |
| 14 | 47 | 1 | 1.38 | .57 |
| 15 | 2 | 1 | 1.35 | .62 |
| 16 | 520 | 1 | 1.27 | .53 |
| 17 | 359 | 1 | 1.43 | .63 |
| 18 | 399 | 1 | 1.40 | .66 |
| 21 | 118 | 1 | 1.27 | .52 |
| 22 | 943 | 1 | 1.31 | .53 |
| 23 | 823 | 1 | 1.27 | .56 |
| 24 | 467 | 1 | 1.32 | .52 |
| 25 | 572 | 1 | 1.31 | .57 |
| 26 | 603 | 1 | 1.32 | .55 |
| 27 | 327 | 1 | 1.41 | .65 |
| 28 | 358 | 1 | 1.52 | .84 |
| 31 | 94 | 1 | 1.29 | .51 |
| 32 | 1090 | 1 | 1.23 | .49 |
| 33 | 325 | 1 | 1.19 | .50 |
| 34 | 817 | 1 | 1.25 | .53 |
| 35 | 417 | 1 | 1.29 | .51 |
| 36 | 475 | 1 | 1.30 | .55 |
| 37 | 283 | 1 | 1.41 | .59 |
| 38 | 409 | 1 | 1.32 | .64 |
| 41 | 182 | 1 | 1.28 | .52 |
| 42 | 1125 | 1 | 1.21 | .45 |
| 43 | 847 | 1 | 1.21 | .42 |
| 44 | 315 | 1 | 1.23 | .53 |
| 45 | 318 | 1 | 1.27 | .55 |
| 46 | 382 | 1 | 1.23 | .56 |
| 47 | 207 | 1 | 1.36 | .56 |
| 48 | 124 | 1 | 1.33 | .51 |
| **total** | **15647** | **1** | 1.29 | .54 |

n= number of teeth of all patients per praxis with median, mean, sd (standard deviation)

Table S2: Collected data of the last visit of the patients regarding tooth mobility

| **Tooth** | **n** | **median** | **mean** | **sd** |
| --- | --- | --- | --- | --- |
| 11 | 818 | 1 | 1.26 | .51 |
| 12 | 622 | 1 | 1.26 | .50 |
| 13 | 328 | 1 | 1.28 | .52 |
| 14 | 445 | 1 | 1.36 | .59 |
| 15 | 494 | 1 | 1.38 | .62 |
| 16 | 323 | 1 | 1.26 | .51 |
| 17 | 410 | 1 | 1.42 | .65 |
| 18 | 124 | 1 | 1.45 | .69 |
| 21 | 893 | 1 | 1.25 | .52 |
| 22 | 762 | 1 | 1.29 | .53 |
| 23 | 423 | 1 | 1.30 | .55 |
| 24 | 555 | 1 | 1.29 | .54 |
| 25 | 535 | 1 | 1.31 | .58 |
| 26 | 297 | 1 | 1.25 | .52 |
| 27 | 331 | 1 | 1.45 | .66 |
| 28 | 88 | 1 | 1.62 | .84 |
| 31 | 1082 | 1 | 1.24 | .49 |
| 32 | 818 | 1 | 1.18 | .46 |
| 33 | 272 | 1 | 1.23 | .49 |
| 34 | 371 | 1 | 1.26 | .56 |
| 35 | 429 | 1 | 1.26 | .50 |
| 36 | 247 | 1 | 1.30 | .55 |
| 37 | 394 | 1 | 1.36 | .59 |
| 38 | 176 | 1 | 1.37 | .64 |
| 41 | 1083 | 1 | 1.25 | .52 |
| 42 | 832 | 1 | 1.19 | .45 |
| 43 | 258 | 1 | 1.17 | .43 |
| 44 | 270 | 1 | 1.20 | .48 |
| 45 | 331 | 1 | 1.28 | .55 |
| 46 | 179 | 1 | 1.24 | .51 |
| 47 | 299 | 1 | 1.40 | .58 |
| 48 | 108 | 1 | 1.36 | .55 |
| **total** | 14597 | **1** | 1.28 | .54 |

n= number of teeth of all patients per praxis with median, mean, sd (standard deviation)

Table S3: Average Bleeding on probing (BOP) of each tooth.

**Tooth = 11**

| **Position** | **n** | **BOP in %** |
| --- | --- | --- |
| ves_dis | 5569 | 19.69 |
| ves_med | 5569 | 8.88 |
| ves_mes | 5569 | 19.15 |
| ora_dis | 5569 | 23.91 |
| ora_med | 5569 | 16.28 |
| ora_mes | 5569 | 21.67 |
| **total** | 33414 | 18.27 |

**Tooth = 12**

| **Position** | **n** | **BOP in %** |
| --- | --- | --- |
| ves_dis | 5468 | 19.69 |
| ves_med | 5468 | 10.71 |
| ves_mes | 5468 | 21.30 |
| ora_dis | 5468 | 23.48 |
| ora_med | 5468 | 16.29 |
| ora_mes | 5468 | 22.38 |
| **total** | 32808 | 18.98 |

**Tooth = 13**

| **Position** | **n** | **BOP in %** |
| --- | --- | --- |
| ves_dis | 5881 | 19.12 |
| ves_med | 5881 | 10.20 |
| ves_mes | 5881 | 22.73 |
| ora_dis | 5881 | 23.80 |
| ora_med | 5881 | 15.47 |
| ora_mes | 5881 | 22.98 |
| **total** | 35286 | 19.05 |

**Tooth = 14**

| **Position** | **n** | **BOP in %** |
| --- | --- | --- |
| ves_dis | 4924 | 21.68 |
| ves_med | 4924 | 10.96 |
| ves_mes | 4924 | 25.97 |
| ora_dis | 4924 | 29.32 |
| ora_med | 4924 | 17.34 |
| ora_mes | 4924 | 27.25 |
| **total** | 29544 | 22.09 |

**Tooth = 15**

| **Position** | **n** | **BOP in %** |
| --- | --- | --- |
| ves_dis | 4902 | 23.54 |
| ves_med | 4902 | 11.40 |
| ves_mes | 4902 | 24.94 |
| ora_dis | 4902 | 31.19 |
| ora_med | 4902 | 19.01 |
| ora_mes | 4902 | 29.19 |
| **total** | 29412 | 23.21 |

**Tooth = 16**

| **Position** | **n** | **BOP in %** |
| --- | --- | --- |
| ves_dis | 4487 | 30.88 |
| ves_med | 4487 | 14.15 |
| ves_mes | 4487 | 30.57 |
| ora_dis | 4487 | 36.99 |
| ora_med | 4487 | 21.93 |
| ora_mes | 4487 | 35.72 |
| **total** | 26922 | 28.37 |

**Tooth = 17**

| **Position** | **n** | **BOP in %** |
| --- | --- | --- |
| ves_dis | 4568 | 32.04 |
| ves_med | 4568 | 17.16 |
| ves_mes | 4568 | 35.24 |
| ora_dis | 4568 | 35.72 |
| ora_med | 4568 | 21.38 |
| ora_mes | 4568 | 37.34 |
| **total** | 27408 | 29.81 |

**Tooth = 18**

| **Position** | **n** | **BOP in %** |
| --- | --- | --- |
| ves_dis | 1382 | 30.31 |
| ves_med | 1382 | 20.40 |
| ves_mes | 1382 | 37.19 |
| ora_dis | 1382 | 34.15 |
| ora_med | 1382 | 22.35 |
| ora_mes | 1382 | 36.68 |
| **total** | 8292 | 30.18 |

**Tooth = 21**

| **Position** | **n** | **BOP in %** |
| --- | --- | --- |
| ves_dis | 5555 | 18.79 |
| ves_med | 5555 | 8.24 |
| ves_mes | 5555 | 18.09 |
| ora_dis | 5555 | 21.99 |
| ora_med | 5555 | 16.38 |
| ora_mes | 5555 | 23.18 |
| **total** | 33330 | 17.78 |

**Tooth = 22**

| **Position** | **n** | **BOP in %** |
| --- | --- | --- |
| ves_dis | 5465 | 19.70 |
| ves_med | 5465 | 9.56 |
| ves_mes | 5465 | 20.76 |
| ora_dis | 5465 | 22.06 |
| ora_med | 5465 | 16.23 |
| ora_mes | 5465 | 23.58 |
| **total** | 32790 | 18.65 |

**Tooth = 23**

| **Position** | **n** | **BOP in %** |
| --- | --- | --- |
| ves_dis | 5887 | 20.04 |
| ves_med | 5887 | 8.73 |
| ves_mes | 5887 | 20.40 |
| ora_dis | 5887 | 24.97 |
| ora_med | 5887 | 16.46 |
| ora_mes | 5887 | 23.71 |
| **total** | 35322 | 19.05 |

**Tooth = 24**

| **Position** | **n** | **BOP in %** |
| --- | --- | --- |
| ves_dis | 4961 | 23.60 |
| ves_med | 4961 | 10.92 |
| ves_mes | 4961 | 24.61 |
| ora_dis | 4961 | 29.75 |
| ora_med | 4961 | 19.02 |
| ora_mes | 4961 | 28.74 |
| **total** | 29766 | 22.77 |

**Tooth = 25**

| **Position** | **n** | **BOP in %** |
| --- | --- | --- |
| ves_dis | 4849 | 25.65 |
| ves_med | 4849 | 11.59 |
| ves_mes | 4849 | 24.14 |
| ora_dis | 4849 | 33.20 |
| ora_med | 4849 | 20.58 |
| ora_mes | 4849 | 29.75 |
| **total** | 29094 | 24.15 |

**Tooth = 26**

| **Position** | **n** | **BOP in %** |
| --- | --- | --- |
| ves_dis | 4396 | 34.03 |
| ves_med | 4396 | 16.21 |
| ves_mes | 4396 | 30.95 |
| ora_dis | 4396 | 37.14 |
| ora_med | 4396 | 22.02 |
| ora_mes | 4396 | 35.23 |
| **total** | 26376 | 29.26 |

**Tooth = 27**

| **Position** | **n** | **BOP in %** |
| --- | --- | --- |
| ves_dis | 4467 | 35.63 |
| ves_med | 4467 | 20.14 |
| ves_mes | 4467 | 34.45 |
| ora_dis | 4467 | 36.93 |
| ora_med | 4467 | 22.47 |
| ora_mes | 4467 | 37.47 |
| **total** | 26802 | 31.18 |

**Tooth = 28**

| **Position** | **n** | **BOP in %** |
| --- | --- | --- |
| ves_dis | 1301 | 32.51 |
| ves_med | 1301 | 21.13 |
| ves_mes | 1301 | 35.97 |
| ora_dis | 1301 | 33.58 |
| ora_med | 1301 | 22.75 |
| ora_mes | 1301 | 36.74 |
| **total** | 7806 | 30.45 |

**Tooth = 31**

| **Position** | **n** | **BOP in %** |
| --- | --- | --- |
| ves_dis | 5825 | 15.45 |
| ves_med | 5825 | 8.65 |
| ves_mes | 5825 | 17.25 |
| ora_dis | 5825 | 17.64 |
| ora_med | 5825 | 12.68 |
| ora_mes | 5825 | 19.24 |
| **total** | 34950 | 15.15 |

**Tooth = 32**

| **Position** | **n** | **BOP in %** |
| --- | --- | --- |
| ves_dis | 5983 | 17.43 |
| ves_med | 5983 | 8.79 |
| ves_mes | 5983 | 16.06 |
| ora_dis | 5983 | 19.42 |
| ora_med | 5983 | 14.08 |
| ora_mes | 5983 | 19.18 |
| **total** | 35898 | 15.83 |

**Tooth = 33**

| **Position** | **n** | **BOP in %** |
| --- | --- | --- |
| ves_dis | 6184 | 17.77 |
| ves_med | 6184 | 8.94 |
| ves_mes | 6184 | 19.11 |
| ora_dis | 6184 | 20.47 |
| ora_med | 6184 | 13.90 |
| ora_mes | 6184 | 20.68 |
| **total** | 37104 | 16.81 |

**Tooth = 34**

| **Position** | **n** | **BOP in %** |
| --- | --- | --- |
| ves_dis | 5694 | 18.63 |
| ves_med | 5694 | 9.58 |
| ves_mes | 5694 | 18.59 |
| ora_dis | 5694 | 25.18 |
| ora_med | 5694 | 16.08 |
| ora_mes | 5694 | 23.39 |
| **total** | 34164 | 18.58 |

**Tooth = 35**

| **Position** | **n** | **BOP in %** |
| --- | --- | --- |
| ves_dis | 5292 | 21.67 |
| ves_med | 5292 | 10.56 |
| ves_mes | 5292 | 21.14 |
| ora_dis | 5292 | 29.04 |
| ora_med | 5292 | 17.80 |
| ora_mes | 5292 | 27.66 |
| **total** | 31752 | 21.31 |

**Tooth = 36**

| **Position** | **n** | **BOP in %** |
| --- | --- | --- |
| ves_dis | 4026 | 30.52 |
| ves_med | 4026 | 17.63 |
| ves_mes | 4026 | 30.79 |
| ora_dis | 4026 | 33.90 |
| ora_med | 4026 | 25.08 |
| ora_mes | 4026 | 34.30 |
| **total** | 24156 | 28.70 |

**Tooth = 37**

| **Position** | **n** | **BOP in %** |
| --- | --- | --- |
| ves_dis | 4611 | 33.52 |
| ves_med | 4611 | 20.84 |
| ves_mes | 4611 | 34.82 |
| ora_dis | 4611 | 32.81 |
| ora_med | 4611 | 22.46 |
| ora_mes | 4611 | 35.82 |
| **total** | 27666 | 30.05 |

**Tooth = 38**

| **Position** | **n** | **BOP in %** |
| --- | --- | --- |
| ves_dis | 1772 | 30.02 |
| ves_med | 1772 | 19.24 |
| ves_mes | 1772 | 33.46 |
| ora_dis | 1772 | 27.59 |
| ora_med | 1772 | 19.80 |
| ora_mes | 1772 | 33.12 |
| **total** | 10632 | 27.21 |

**Tooth = 41**

| **Position** | **n** | **BOP in %** |
| --- | --- | --- |
| ves_dis | 5810 | 17.88 |
| ves_med | 5810 | 9.87 |
| ves_mes | 5810 | 18.46 |
| ora_dis | 5810 | 18.82 |
| ora_med | 5810 | 12.85 |
| ora_mes | 5810 | 18.08 |
| **total** | 34860 | 16.00 |

**Tooth = 42**

| **Position** | **n** | **BOP in %** |
| --- | --- | --- |
| ves_dis | 5952 | 19.43 |
| ves_med | 5952 | 10.21 |
| ves_mes | 5952 | 18.12 |
| ora_dis | 5952 | 20.74 |
| ora_med | 5952 | 13.72 |
| ora_mes | 5952 | 18.83 |
| **total** | 35712 | 16.84 |

**Tooth = 43**

| **Position** | **n** | **BOP in %** |
| --- | --- | --- |
| ves_dis | 6174 | 17.08 |
| ves_med | 6174 | 10.25 |
| ves_mes | 6174 | 20.92 |
| ora_dis | 6174 | 21.97 |
| ora_med | 6174 | 14.02 |
| ora_mes | 6174 | 21.26 |
| **total** | 37044 | 17.71 |

**Tooth = 44**

| **Position** | **n** | **BOP in %** |
| --- | --- | --- |
| ves_dis | 5733 | 18.59 |
| ves_med | 5733 | 10.34 |
| ves_mes | 5733 | 21.52 |
| ora_dis | 5733 | 26.67 |
| ora_med | 5733 | 15.10 |
| ora_mes | 5733 | 24.21 |
| **total** | 34398 | 19.40 |

**Tooth = 45**

| **Position** | **n** | **BOP in %** |
| --- | --- | --- |
| ves_dis | 5362 | 22.06 |
| ves_med | 5362 | 10.92 |
| ves_mes | 5362 | 22.06 |
| ora_dis | 5362 | 28.36 |
| ora_med | 5362 | 16.74 |
| ora_mes | 5362 | 25.73 |
| **total** | 32172 | 20.98 |

**Tooth = 46**

| **Position** | **n** | **BOP in %** |
| --- | --- | --- |
| ves_dis | 4124 | 29.60 |
| ves_med | 4124 | 16.48 |
| ves_mes | 4124 | 29.77 |
| ora_dis | 4124 | 34.69 |
| ora_med | 4124 | 22.59 |
| ora_mes | 4124 | 33.22 |
| **total** | 24744 | 27.73 |

**Tooth = 47**

| **Position** | **n** | **BOP in %** |
| --- | --- | --- |
| ves_dis | 4738 | 32.35 |
| ves_med | 4738 | 19.33 |
| ves_mes | 4738 | 34.36 |
| ora_dis | 4738 | 33.28 |
| ora_med | 4738 | 24.54 |
| ora_mes | 4738 | 35.66 |
| **total** | 28428 | 29.92 |

**Tooth = 48**

| **Position** | **n** | **BOP in %** |
| --- | --- | --- |
| ves_dis | 1821 | 30.20 |
| ves_med | 1821 | 20.31 |
| ves_mes | 1821 | 34.70 |
| ora_dis | 1821 | 31.30 |
| ora_med | 1821 | 22.84 |
| ora_mes | 1821 | 35.03 |
| **total** | 10926 | 29.06 |
